# The germinal center-tertiary lymphoid structure after neoadjuvant chemo-immunotherapy for locally advanced lung squamous cell carcinoma can predict the disease progression

**DOI:** 10.3389/fimmu.2025.1579840

**Published:** 2025-09-12

**Authors:** Shuang Li, Ping Zhou, Yan Huang, Min Chen, Chan Yang, Lili Jiang

**Affiliations:** ^1^ Department of Pathology, West China Hospital, Sichuan University, Chengdu, China; ^2^ Department of Pathology, The First People's Hospital of Yunnan Province, Yunnan, Kunming, China; ^3^ Department of Medical Oncology, Cancer Center, West China Hospital, Sichuan University, Chengdu, Sichuan, China; ^4^ College of Physical Education and Health Science, Yi bin University, Yi bin, Sichuan, China

**Keywords:** neoadjuvant chemoimmunotherapy, lung squamous cell carcinoma, prognostic biomarkers, tertiary lymphoid structures (TLSs), neoadjuvant chemotherapy

## Abstract

**Background:**

The tertiary lymphoid structures (TLSs) are the anti-tumor immune hubs in the tumor microenvironment. The germinal center (GC) (a marker of maturation) and spatial distribution of TLS may determine the responsiveness of immunotherapy. However, the regulatory mechanism of neoadjuvant chemotherapy (NACT) and combined immunotherapy (NACT-IO) on the dynamic remodeling of TLS has not been elucidated.

**Methods:**

The NACT-IO group (72 patients), NACT group (50 patients), UT group (50 patients, un-neoadjuvant therapy) were included. Multiple immunofluorescence (mIF) was used to analyze the difference of microenvironment in paired samples (the same case) pre and post neoadjuvant therapy. To further analyze the effect of treatment on the maturity and spatial distribution pattern of TLS (within/outside tumor bed) in postoperative samples, and to establish a quantitative method of TLS based on hot spot area to evaluate its prognostic value.

**Results:**

Spatial heterogeneity analysis that the density of total TLSs (t-TLSs) and GC-positive TLSs (GC-TLSs) in the tumor bed of NACT (*p*<0.01, *p*<0.01) group and NACT-IO (*p*<0.001, *p*<0.001) group were significantly higher than that outside the tumor bed. Compared with the UT group, NACT and NACT-IO significantly increased the density of t-TLSs (*p*<0.01, *p*<0.001) and GC-TLSs (*p*<0.01, *p*<0.01) in the tumor bed. In addition, there was an inverted U-shaped correlation between GC-TLS and treatment cycle: the density of GC-TLSs reaches the peak value after receiving two or less (≤ 2) cycles of NACT and NACT-IO, and decreased significantly after receiving more than two (> 2) cycles of NACT and NACT-IO (*p*<0.05). Multivariate Cox regression model confirmed that low GC-TLS burden (≤2/20×HPF) within tumor bed hotspots (HR = 3.99, 95%CI=1.10-14.5, *p* = 0.036) was superior to the traditional prognostic factor of pathological remission in ≤ 2-cycles of NACT-IO subgroup (HR = 3.44, 95%CI=1.03-11.47, *p* = 0.044), and became the strongest independent factor for predicting disease free survival (DFS).

**Conclusions:**

This study reveals for the first time that NACT and NACT-IO enhance anti-tumor efficacy through multidimensional (abundance, spatial distribution and maturity) dynamic remodeling of TLS, and proposes the short course of ≤ 2 cycles of NACT-IO can maximize the prognostic value of GC-TLS, providing key evidence for optimizing the treatment ‘ time window ‘.

## Introduction

Lung cancer remains the leading cause of cancer-related deaths worldwide, with non-small cell lung cancer (NSCLC) accounting for 85% (1). Squamous cell carcinoma (SCC) is the second most common type of NSCLC (2). Chemotherapy is the standard treatment in the perioperative period, but the efficacy is limited (3). The preoperative neoadjuvant therapy scheme can reduce the size of the primary tumor to further facilitate surgical operation and improve postoperative prognosis (4). In recent years, immunotherapy has emerged as a promising new approach. Additionally, the neoadjuvant chemotherapy combined with immunotherapy could significantly improve the pathological remission rate and prolong the event-free survival (EFS) (5). Nevertheless, some patients still face a high risk of recurrence, suggesting that there is still an unmet need for us to explore the identification and predictive markers of relapse-prone populations.

The efficacy of immunotherapy can be attributed to the highlighted impact of the tumor immune microenvironment (TIME) (6), which refers to the cellular environment around tumor cells (7). Immune cells play a central role in this environment, including but not limited to: T lymphocytes, B lymphocytes, natural killer cells, bone marrow-derived suppressor cells, dendritic cells, macrophages (8). The tertiary lymphoid structure (TLS) is an ectopic lymphoid tissue dominated by B-cell follicles and T-cell regions in chronic inflammation or tumor microenvironment (9). In recent years, a large number of studies have shown that TLS has a positive anti-tumor effect in different cancers, especially when it is in the mature stage (10). According to its histological characteristics and functional activity, TLS can be divided into immature stage with only lymphocyte aggregation, lack of germinal center (GC) and clear partition, and mature stage with GC, follicular dendritic cell (FDC) network, high endothelial venule (HEV) and clear B/T cell partition (10, 11). The tight interaction between B cells and T cells in TLSs is related to the improvement of cancer survival rate and hot tumor environment (12). Within TLS, B cells undergo GC reactions to produce high-affinity tumor-specific antibodies. These antibodies form immune complexes by engaging Fcγ receptor (FcγR)-expressing dendritic cells (DCs) and macrophages in the extrafollicular zones, thereby triggering the antigen cross-presentation cascade. Through MHC class I molecules, tumor-derived antigens are presented to CD8^+^T lymphocytes, driving their differentiation into cytotoxic effector cells capable of direct tumor cell elimination. Concurrently, CD4^+^T cells secrete key cytokines (such as IL - 21, IFN-γ) with the help of B cell-dependent antigen presentation, which synergistically enhance the anti-tumor immune response (9, 13). In addition to the characteristics of abundance and maturity of TLS, the effect of its location distribution on the prognosis of colorectal cancer, hepatocellular carcinoma, breast cancer and esophageal squamous cell carcinoma has also been explored (14–19).

TLS creates a microenvironment that is sensitive to immunotherapy (9) and can predict favorable responses to immune checkpoint blockade in a variety of malignant tumors (19, 20). Intriguingly, Helmink et al. demonstrated that immune checkpoint inhibitors (ICIs) coordinate the formation and maturation of TLS by reprogramming chemokine networks (CXCL9/10/13) and lymphatic induction signals (LT-α/β) (21). Chemotherapy is also a conventional anti-tumor treatment, and its effect on TLS function is still poorly understood. The results of SILIA K et al. lung squamous cell carcinoma showed that corticosteroids (the main pillar of chemotherapy regimens) disrupted TLS maturation by inhibiting lymphatic-induced LT-α/β signaling, thereby eliminating GC formation and weakening CD8^+^T cell infiltration (11). However, it is worth noting that lu zhang et al. used a bladder cohort to reveal the synergistic effect of ICIs and chemotherapeutic drugs on enhancing TLS maturity (22). This study used a 172 SCC cohort to analyze the interaction between TLS maturity, spatial topology, and treatment regimen. By combining multiple spatial phenotypes with longitudinal survival analysis, our objectives are: (1) to explore the differential regulation of chemotherapy and ICIs on the spatial distribution and maturity of TLS, (2) to explore the characteristics of TLS as a predictive value of disease progression after SCC receiving NACT and NACT-IO, and (3) to optimize the treatment of TLS-driven immunity.

## Materials and methods

### Patient collection and tissues specimens

The locally advanced SCC samples that underwent surgical resection after neoadjuvant therapy in West China Hospital of Sichuan University from January 2018 and December 2022 were collected. We included patients who received NACT (50 cases) and NACT-IO (72 cases) and matched 50 cases of surgical resection SCC samples that did not receive any neoadjuvant therapy (UT group). Preoperative paired biopsy samples of SCC patients are difficult to systematically evaluate the integrity of TLS due to tissue fragmentation. Therefore, we attempted to preliminarily quantify the dynamic changes of lymphocyte subsets in TIME before and after treatment by multiple immunofluorescence staining. Among the 172 patients in the NACT-IO group and the NACT group, we screened 64 cases with paired biopsy specimens before and after treatment. In order to ensure the objectivity of the evaluation, 64 paired samples of TIME were evaluated by two independent senior pathologists, considering the samples with rich immune cells, clear tumor tissue, sufficient interstitial tissue and complete clinical data. Finally, 10 cases (4 cases with NACT-IO, 6 cases with NACT) that met the strict quality control were included for subsequent analysis. Clinical data were obtained from the medical records, including gender, age, smoking history, tumor site and follow-up data. The follow-up period ended in April 2, 2024.

### Multiple immunofluorescence and immunohistochemistry

Paraffin sections were baked at 62 °C for 60 minutes, followed by xylene gradient dewaxing and ethanol gradient hydration. Antigen repair was performed by high-pressure method, the first round was repaired with a pH 9.0 repair solution, and the subsequent high-pressure treatment was repeated with a pH 6.0 repair solution before each round of staining to eliminate antibody cross-reaction. Each round of staining process included TBST washing and blocking with bovine and sheep mixed serum, followed by primary antibody incubation (antibodies and dilution ratios were as follows: CD4, CD8, CD19, PD1, and then incubated with secondary antibodies (goat anti-mouse/rabbit mixed secondary antibody, 37 °C 10 minutes). Finally, Opal fluorescent dye was used for color development (Opal 520/570/620/690/780, room temperature 5 – 10 minutes). DAIP was used for re-staining nucleus, and finally the tablets were sealed with anti-quenching sealing agent. The stained sections were scanned using the TissueFAXS SL panoramic tissue cell imaging system. Analysis was performed using StrataQuest analysis software (version 7.1.129). Firstly, tumor cells, stromal cells and lymphocyte subsets were distinguished based on DAPI nuclear staining and morphological features (area, roundness). Then the fluorescence intensity threshold of each marker (CD4/CD8/CD19/PD1) was set. Finally, the proportion of positive cells was calculated.

The formalin-fixed paraffin-embedded (FFPE) tissues of 172 patients with SCC were serially sectioned (4μm thickness) and baked at 65°C for 1 hour. The sections were subjected to xylene gradient dewaxing and ethanol gradient hydration, rinsed with double distilled water, and incubated with 3% H_2_O_2_ at room temperature for 25 minutes to block endogenous peroxidase activity. Antigen repair was performed using pH 6.0 citrate buffer, boiled under high pressure for 3 minutes. CD8, CD19, CD21, CD23 and D2 – 40 were incubated at 37°C for 1 hour. Subsequently, HRP-labeled goat anti-mouse/rabbit universal secondary antibody (ready-to-use, ZSGB-Bio, Cat#PV-9000) was incubated at 45°C for 30 minutes. Finally, DAB staining was performed for 5 minutes and re-stained with hematoxylin. The digital pathological images were collected by KFBIO automatic scanning system, and quantitative analysis was performed by K-Viewer software (v2.3.1). All IHC was performed on a Leica Bond-Max or Roche Ventana system. Details of all antibodies and batch verification procedures are available in **Supplementary Table S1**.

### Assessment of pathological response

Two pathologists with advanced qualifications assessed the pathological remission of samples from NACT-IO group and NACT group. Throughout this process, they were not informed of the specific details and objectives of this study. CPR is defined as complete pathological response, indicating the absence of residual surviving tumor cells (RVT). MPR and PPR are defined as major pathological response (0%<RVT<10%) and partial pathological response (RVT ≥10%) respectively. In this study, we combined cases with residual tumor ≤ 10%, including CPR and MPR, with PPR to form a binary stratification.

### Assessment of the TLS

The spatial niche of TLSs is divided into (1) TLSs within the tumor bed (the area where the original tumor tissue exists), including TLSs in the tumor (residual tumor tissue) and TLSs in the stroma; (2) TLSs outside the tumor bed (2mm area outside the edge of the tumor bed) (**Figure 2A**). TLSs are divided into three mature stages: early TLS(E-TLS), primary follicle-like TLS (PFL-TLS) and secondary follicle-like TLS (SFL-TLS) (16). E-TLS is a loosely clustered lymphocyte population, lacking a clear follicular structure and GC. CD19^+^B cells are scattered and non-aggregated, CD8^+^T cells do not form T cell regions, and there is no follicular dendritic cell (FDC) network and high endothelial venule (HEV). PFL-TLS has a preliminary B cell follicular structure, but there is no GC, and the T cell region begins to differentiate. CD19^+^B cells gathered into clusters, CD8^+^T cells formed a peripheral T cell band, and CD21^+^FDC formed a preliminary network structure. SFL-TLS is a fully mature stage, and CD23 is a marker of functional active GC. Total TLS (t-TLS) includes E-TLS, PFL-TLS and SFL-TLS. Among them, SFL-TLS was counted as GC-TLS (**Figure 2B**).

### Assessment of the CD8^+^T cells within in GC-TLSs/outside TLSs

The formation and maturation of TLS (GC differentiation) are dynamically regulated by the treatment cycle. As the core effector cells of anti-tumor immunity, the spatial distribution heterogeneity of CD8^+^ T cells inside and outside TLS may further reflect the reprogramming of immune response pattern by treatment. The spatial distribution of CD8**^+^** T cells was divided into regions located within in GC-TLSs and outside TLSs. We used KF-view software to count the density of CD8**^+^** T cells in any 10 target areas with an area of more than 1 mm^2^, and calculated the average value (**Figures 2C-F**).

### Inter-observer agreement analysis

Two blinded thoracic pathologists independently selected randomly 100 samples assessed the t-TLS/GC-TLS burden within tumor bed hotspots (binary: ≤3/>3/20×HPF, ≤2/>2/20×HPF). Detailed data are provided in **Supplementary Table S2**.

### Statistical analysis

All statistical analysis and visualization were performed in the R language environment (RStudio 4.2.3). There was a significant difference when *p* < 0.05.

#### Data distribution and paired sample analysis

Analysis of clinicopathological features: The categorical variables such as clinicopathological features of 172 patients were analyzed by chi-square test or Fisher ‘s exact test.

Paired sample analysis: For the dynamic changes of lymphocyte subsets in TIME of 10 paired samples from the same patient before and after treatment, the Wilcoxon Signed-Rank Test was used to evaluate the difference before and after treatment.

#### Comparison of continuous variables between groups

Cross-group comparison (NACT group vs. NACT-IO group vs. UT group): For continuous variables such as TLS density (t-TLS, GC-TLS) and CD8**^+^** T cell count, Kruskal-Wallis H test (Kruskal-Wallis Test) was used.

Subgroup analysis: treatment cycle stratification (≤ 2 cycles vs. > 2 cycles) and pathological remission stratification (CPR/MPR cases vs. PPR cases), using Mann-Whitney U test (Wilcoxon Rank-Sum Test).

#### Survival analysis and prognostic model

Survival grouping: Patients were divided into high/low (t-/GC-) TLS density group with median cut-off value. Kaplan-Meier method (Log-rank test) was used to evaluate the difference of disease-free survival (DFS), and the survival curve was visualized by ggsurvplot package (v 0.4.9). DFS was defined as the time from surgical resection to disease recurrence, death (for any reason), or loss to follow-up.

Prognostic factor screening: The purpose of this study was to screen TLS-related prognostic factors with high clinical applicability and repeatability of evaluation principles, and to implement a grouping strategy based on the number of t-TLS in hot spots (≤ 3/20×HPF vs. >3/20×HPF) and the number of GC-TLS in hot spots (≤ 2/20×HPF vs. >2/20×HPF), rather than using the median of TLS as a threshold. Univariate Cox Regression model was used to screen potential prognostic factors, and Multivariate Cox Regression model was constructed to correct confounding factors such as age, gender, and smoking history. The association between the number of t-/GC-TLS in hot spots and pathological remission grading and DFS was evaluated, and independent prognostic factors were screened.

The inter-observer agreement was quantified using Cohen’s kappa (κ), with 95% confidence intervals estimated via bias-corrected and accelerated (BCa) bootstrap resampling (1000 iterations).

## Results

### Patient characteristics

The clinicopathological characteristics are summarized in **Table 1**, with a mean age of 57.4 ± 7.3 years. There were no significant differences in age, sex, smoking history, treatment cycles, or N stage between NACT-IO group and NACT group. 52 cases (52/72, 72.2%) obtained CPR/MPR and 20 cases (20/72, 27.8%) obtained PPR in NACT-IO group. Conversely, in the NACT group, only 11 cases (11/50, 22%) obtained CPR/MPR and 39 cases (39/50, 78.0%) obtained MPR. Detailed data are provided in **Supplementary Table S3**.

**Table 1:** The clinicopathological characteristics of 172 patients in all groups.

Characteristics		NACT-IO n=72	NACT n=50	UT n=50	*p*
Age					0.615
<57		31 (43.1%)	26 (52.0%)	24 (48.0%)	
≥57		41 (56.9%)	24 (48.0%)	26 (52.0%)	
Sex					0.885
female		4 (5.56%)	1 (2.00%)	2 (4.00%)	
male		68 (94.4%)	49 (98.0%)	48 (96.0%)	
Smoke history					0.477
no		18 (25.0%)	10 (20.0%)	8 (16.0%)	
yes		54 (75.0%)	40 (80.0%)	42 (84.0%)	
Cycles					0.370
>2		34 (47.2%)	18 (36.0%)	——	
≤2		38 (52.8%)	32 (64.0%)	——	
N stage					0.052
pN0	pN0	44 (61.1%)	25 (50.0%)	30 (60.0%)	
	atN0	9 (12.5%)			
pN1		9 (12.5%)	10 (20.0%)	12 (24.0%)	
pN2		10 (13.9%)	15 (30.0%)	8 (16.0%)	
Pathologic response					<0.001
CPR/MPR		52 (72.2%)	11 (22.0%)	——	
PPR		20 (27.8%)	39 (78.0%)	——	

primary N0 (pN0) refers to no tumor metastasis and treatment response in lymph nodes; after neoadjuvant treatment N0 (atN0) refers to the presence of therapeutic response in lymph nodes, but no residual tumor.

### B cells recruitment may be the common mechanism of chemotherapy and immunotherapy

**Figure 1:**
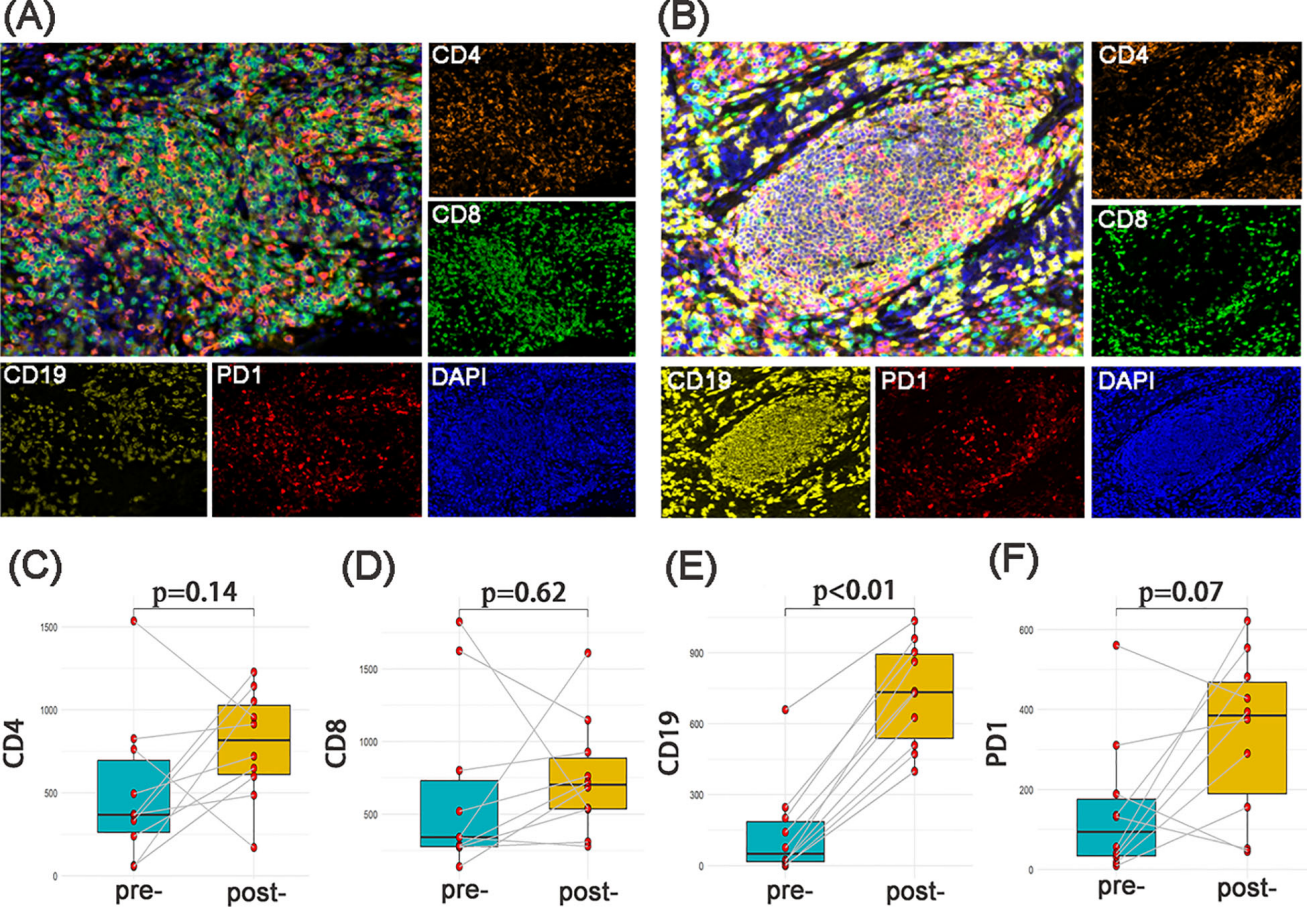
The changes of immune microenvironment between SCC samples pre and post neoadjuvant therapy (n=10). The multi-channel mIF(upper left) and single-channel mIF of lymphocyte subsets in 10 paired SCC samples pre **(A)** and post **(B)** neoadjuvant therapy (4 cases of NACT-IO, 6 cases of NACT) were showed. Compared with SCC samples pre-treatment (n=10), CD19^+^B cells were significantly increased in SCC samples post-treatment (n=10) **(E)**. There was no significant difference of CD4^+^T cells **(C)**, CD8^+^T cells **(D)** and PD - 1^+^lymphocytes **(F)** pre- and post- neoadjuvant therapy. The p-value was obtained by Wilcoxon signed rank sum test. *p*< 0.05 was considered statistically significant. **(A)** magnification×200, **(B)** magnification×200.

It is worth noting that despite the heterogeneity of treatment strategies (NACT vs. NACT-IO), the density of CD19^+^ B cell in post- NACT-IO/NACT were significantly higher (729.39 ± 217.66 cells/mm², *p*<0.01) than that pre- NACT-IO/NACT (n=10) (**Figure 1E**). However, there was no such change in CD4^+^T cell (913.04 ± 243.03 cells/mm², *p* = 0.14), CD8^+^T cell (541.59 ± 483.79 cells/mm², *p* = 0.62) and PD1^+^ lymphocyte (394.75 ± 129.98 cells/mm², *p* = 0.07) (**Figures 1C, D, F**).

**Figure 2:**
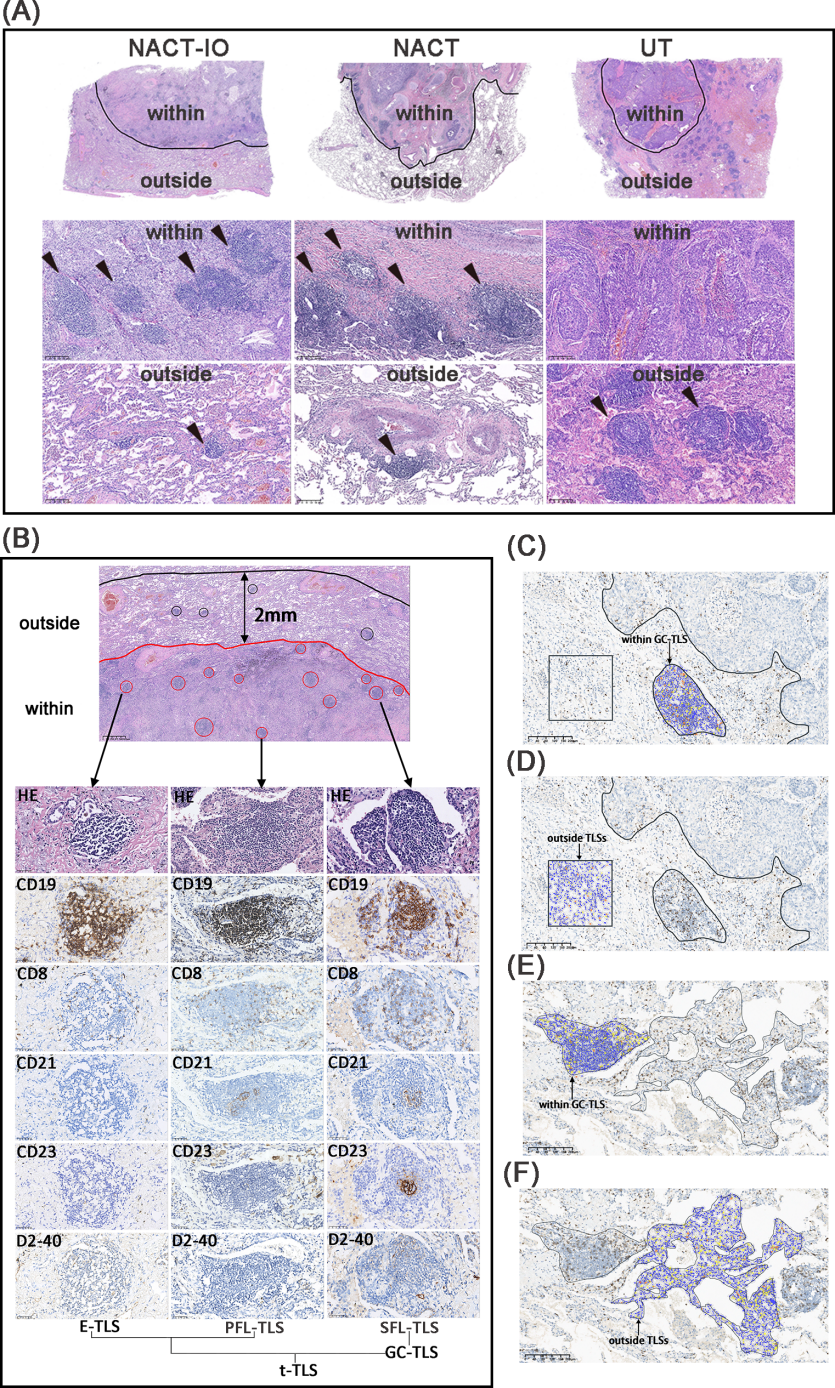
The niche distribution characteristics of TLSs and CD8^+^ T cell location distribution. The TLSs located the within- and outside- regions of tumor bed in NACT-IO group (left), NACT group (middle) and UT group (right) **(A)**. The TLSs at different developmental stages, including E-TLS, PFL-TLS and SFL-TLS. The E-TLS was characterized by CD19^+^, CD8^+^, CD21^—^, CD23^—^ and D2 - 40^—^. The PFL-TLS was characterized by CD19^+^, CD8^+^, CD21^+^, CD23^—^ and D2 - 40^+^. The SFL-TLS was characterized by CD19^+^, CD8^+^, CD21^+^, CD23^+^ and D2 - 40^+^. E-TLS, PFL-TLS and SFL-TLS were counted as t-TLS. Among them, SFL-TLS is GC-TLS **(B)**. Quantitative assessment of CD8^+^T cell density in the tumor bed, including areas within GC-TLS (black arrow, oval area) and areas outside TLSs (black arrow, square area) **(C, D)**. Quantitative assessment of CD8^+^ T cell density outside the tumor bed, including areas within GC-TLS (black arrow, oval area) and areas outside TLSs (black arrow, irregular area). **(C, D)**. **(C–F)** magnification×100.

### NACT and NACT-IO induced TLSs formation and differentiation into mature phenotype (GC formation) and directed TLSs recruitment to the tumor bed (antigen-rich region)

**Figure 3:**
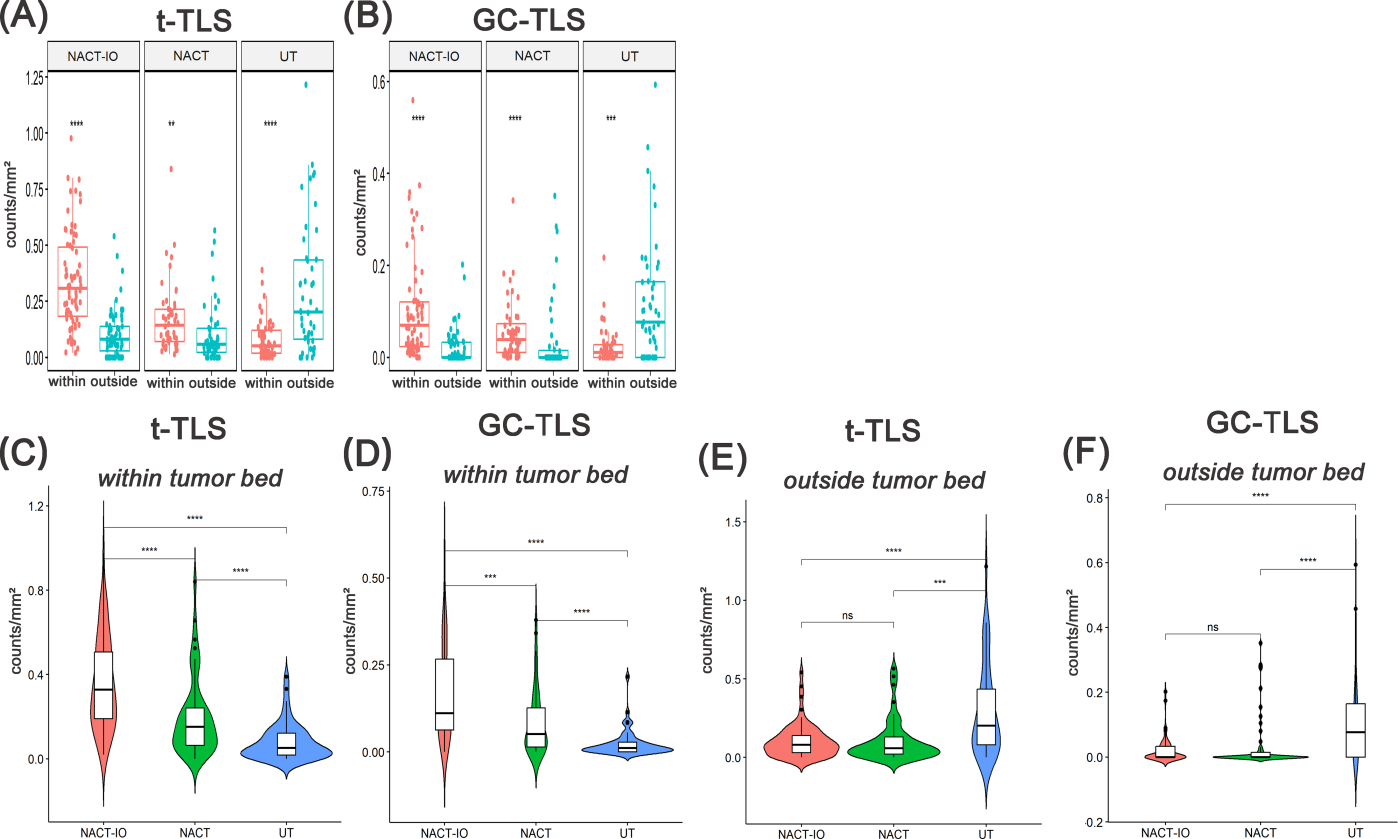
Analysis of the spatial distribution of t-/GC-TLSs in NACT-IO group(n=72), NACT group(n=50) and UT group(n=50) and between groups. The density difference of t-TLS **(A)** and GC-TLS **(B)** in the within and outside regions of the tumor bed in the NACT-IO group, the NACT group and the UT group. The differences of t-TLS **(C)** and GC-TLS **(D)** within the tumor bed were compared among NACT-IO group, NACT group and UT group. The differences of t-TLS **(E)** and GC-TLS **(F)** outside the tumor bed were compared among NACT-IO group, NACT group and UT group. The *p* value was obtained by Wilcoxon rank sum test (* * means *p* < 0.01; * * * denotes *p* < 0.001; *ns* means no statistical difference).

Firstly, the differences of t-/GC-TLSs within the tumor bed and outside the tumor bed in the three groups were analyzed. The t-TLSs within the tumor bed of the NACT-IO group (within/outside: 0.31 ± 0.21 *vs* 0.08 ± 0.11 t-TLSs/mm², *p*<0.001) and NACT group (within/outside: 0.14 ± 0.15 *vs* 0.06 ± 0.14 t-TLSs/mm², *p*<0.01) were significantly higher than that outside the tumor bed, while the UT group (within/outside: 0.05 ± 0.09 *vs* 0.20 ± 0.29 t-TLSs/mm², *p*<0.01) was the opposite (**Figure 3A**). Similarly, The GC-TLSs within the tumor bed of the NACT group (within/outside: 0.04 ± 0.06 *vs* 0.00 ± 0.08 GC-TLSs/mm², *p*<0.001) and NACT-IO group (within/outside: 0.07 ± 0.11 *vs* 0.00 ± 0.04 GC-TLSs/mm², *p*<0.001) were significantly higher than that outside the tumor bed, while the UT group (within/outside: 0.01 ± 0.04 *vs* 0.08 ± 0.13 GC-TLSs/mm², *p*<0.001) was the opposite (**Figure 3B**).

Then, the differences of t-/GC-TLSs in the same spatial location among the three groups were analyzed. The t-TLSs within the tumor bed of the NACT-IO group and NACT group were significantly higher than that of the UT group (NACT-IO group/UT group: 0.20 ± 0.29 *vs* 0.06 ± 0.14 t-TLSs/mm², *p*<0.01; NACT group/UT group: 0.14 ± 0.15 *vs* 0.05 ± 0.09 t-TLSs/mm², *p*<0.01) (**Figure 3C**). The GC-TLSs within the tumor bed of the NACT-IO group and NACT-IO group were significantly higher than that of the UT group (NACT-IO group/UT group: 0.08 ± 0.13 *vs* 0.00 ± 0.09 GC-TLSs/mm², *p*<0.01; NACT group/UT group: 0.04 ± 0.06 *vs* 0.01 ± 0.04 GC-TLSs/mm², *p*<0.01) (**Figure 3D**). However, the t-TLSs outside the tumor bed of the NACT-IO group and NACT group were significantly lower than that of the UT group (NACT-IO group/UT group: 0.08 ± 0.11 *vs* 0.20 ± 0.29 t-TLSs/mm²; NACT group/UT group: 0.06 ± 0.14 *vs* 0.20 ± 0.29 t-TLSs/mm², *p*<0.01) (**Figure 3E**). The GC-TLSs outside the tumor bed of the NACT-IO group and NACT-IO group were significantly lower than that of the UT group (NACT-IO group/UT group: 0.00 ± 0.04 *vs* 0.08 ± 0.13 GC-TLSs/mm², *p*<0.01; NACT group/UT group: 0.00 ± 0.08 *vs* 0.08 ± 0.13 GC-TLSs/mm², *p*<0.01) (**Figure 3F**).

### NACT-IO and NACT inhibited the induction of TLSs in a time-dependent manner

**Figure 4:**
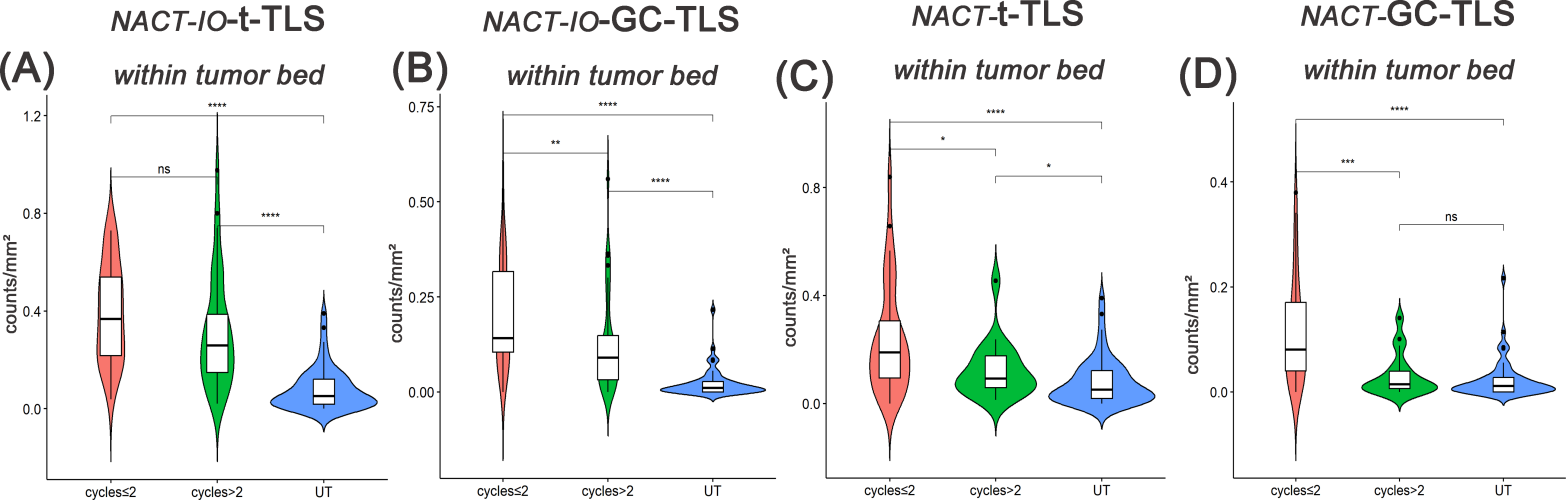
Differences in t-/GC-TLSs located in the tumor bed between UT group (baseline) and samples after receiving two or less(≤2) or more than two(>2) cycles of NACT-IO/NACT. Differences in t-TLSs **(A)** and GC-TLSs **(B)** located in the tumor bed between UT group (baseline) and samples after ≤2(n=38)/>2(n=34) cycles of NACT-IO. Differences in t-TLSs **(C)** and GC-TLSs **(D)** located in the tumor bed between UT group (baseline) and samples after ≤2(n=32)/>2(n=18) cycles of NACT. The p value was obtained by Wilcoxon rank sum test (* * means *p* < 0.01; * * * denotes *p* < 0.001; *ns* means no statistical difference).

The differences of t-/GC-TLSs in NACT-IO group among patients receiving two or less (≤ 2) (n=38) or more than two (> 2) (n=34) treatment cycles were analyzed. The t-/GC-TLSs in the tumor bed was significantly higher than the baseline level (UT group), regardless of receiving ≤ 2 or > 2 cycles of NACT-IO (*p*<0.01, *p*<0.001) (**Figures 4A, B**). Among them, after receiving > 2 cycles of NACT-IO, t-/GC-TLS in the tumor bed decreased, but the decrease of GC-TLSs was significant (*p*<0.01) (**Figure 4B**). Similarly, the t-/GC-TLSs in the tumor bed were significantly higher than the baseline level (UT group) after receiving ≤ 2 (n=32) cycles of NACT (*p*<0.001) (**Figures 4C, D**). However, after receiving > 2 (n=18) cycles of NACT, t-/GC-TLS in the tumor bed both significantly decreased (*p*<0.05, *p*<0.001) **(Figures 4C, D**). Among them, GC-TLSs in the tumor bed even decreased to no significant difference from the baseline in patients receiving ≤ 2 cycles of NACT.

### Long-term NACT may lead to depletion extravasation of CD8^+^T cells in GC-TLS, and combined immunotherapy maintains the efficacy of GC-TLS by reshaping the spatial distribution of CD8^+^T cells

**Figure 5:**
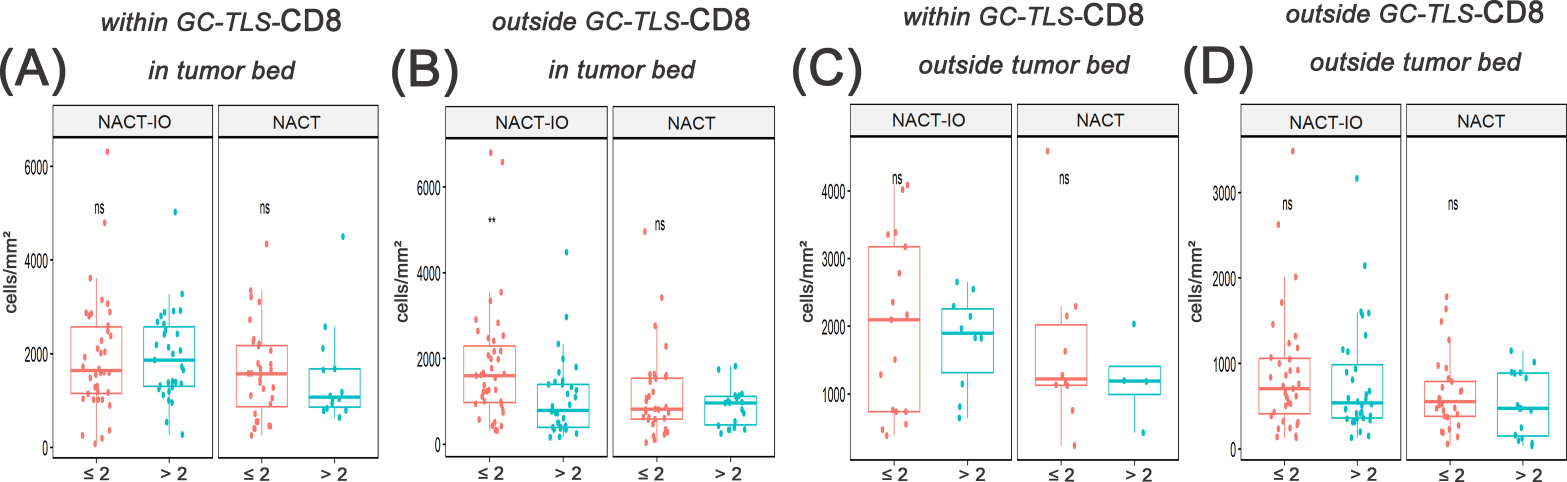
The correlation between the migration and enrichment pattern of CD8^+^T cells and receiving ≤ 2 or > 2 cycles of NACT-IO/NACT was analyzed. The difference of CD8^+^T cells within GC-TLSs in tumor bed between patients receiving ≤2(n=38)/>2(n=34) cycles of NACT-IO(A, left) and ≤2(n=32)/>2(n=18) cycles of NACT(A, right). The difference of CD8^+^T cells outside TLSs in tumor bed between patients receiving ≤2/>2 cycles of NACT-IO(B, left)/NACT(B, right). The difference of CD8^+^T cells within GC-TLSs outside tumor bed between patients receiving ≤2/>2 cycles of NACT-IO(C, left)/NACT(C, right). The difference of CD8^+^T cells outside TLSs outside tumor bed between patients receiving ≤2/>2 cycles of NACT-IO(D, left)/NACT(D, right). The p value was obtained by Wilcoxon rank sum test (* * means *p* < 0.01; * * * denotes *p* < 0.001; *ns* means no statistical difference).

The differences of CD8^+^T cells with different spatial distribution in NACT-IO group/NACT group in patients receiving different treatment cycles were analyzed. After receiving > 2 cycles of NACT-IO, it was no significant change in the infiltration of CD8 (T cells) within the GC-TLS region (**Figure 5A**, **left**), while the infiltration of CD8^+^T cells in the outer region of TLSs decreased significantly (*p*<0.05) (**Figure 5B**, **left**). Differently, after receiving > 2 cycles of NACT, the infiltration of CD8^+^T cells in GC-TLS decreased (**Figure 5A**, **right**), while the infiltration of CD8^+^T cells in the outer region of TLSs increased (**Figure 5B**, **right**).

### The induction effect of NACT on the formation and maturation of TLS may be positively correlated with tumor tissue load, but the combination of immunotherapy makes TLS compensatory proliferation

**Figure 6:**
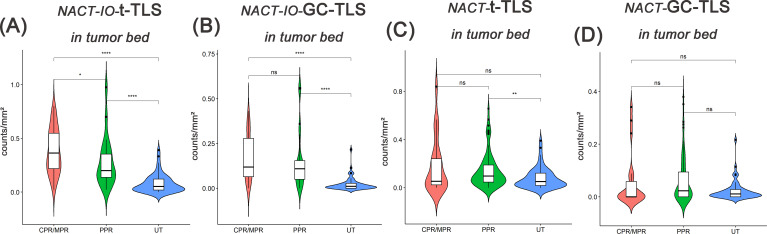
The difference of t-TLSs/GC-TLSs within tumor bed in different pathological remission cases between UT group (baseline) and NACT-IO group/NACT group. The difference of t-TLSs **(A)** /GC-TLSs **(B)** in tumor bed between CPR/MPR(n=52) and PPR(n=20) cases in UT group (baseline) and NACT-IO group. The difference of t-TLSs **(C)** /GC-TLSs **(D)** in tumor bed between CPR/MPR(n=11) and PPR(n=39) cases in UT group (baseline) and NACT group. The p value was obtained by Wilcoxon rank sum test (* * means *p* < 0.01; * * * denotes *p* < 0.001; *ns* means no statistical difference).

The relationship between t-/GC-TLS in tumor bed and different pathological remission cases (CPR/MPR vs. PPR) in NACT-IO group/NACT group was analyzed. In the NACT group, t-TLS in the tumor bed increased in the PPR cases (n=39) compared with the baseline (UT group) (**Figure 6C**), while the t-/GC-TLSs in the tumor bed were no significant change in the CPR/MPR cases (n=11) (**Figures 6C, D**). Moreover, after combined immunotherapy (NACT-IO group), the t-/GC-TLSs in the tumor bed of CPR/MPR cases (n=52) were higher than those of UT and PPR cases (n=20) (**Figures 6A, B**), and t-TLS was a significant advantage (*p*<0.001, *p*<0.05) (**Figure 6A**).

### Two cycles of NACT-IO may be a time window period for immune activation, and GC-TLS in the tumor bed can predict disease progression

**Figure 7:**
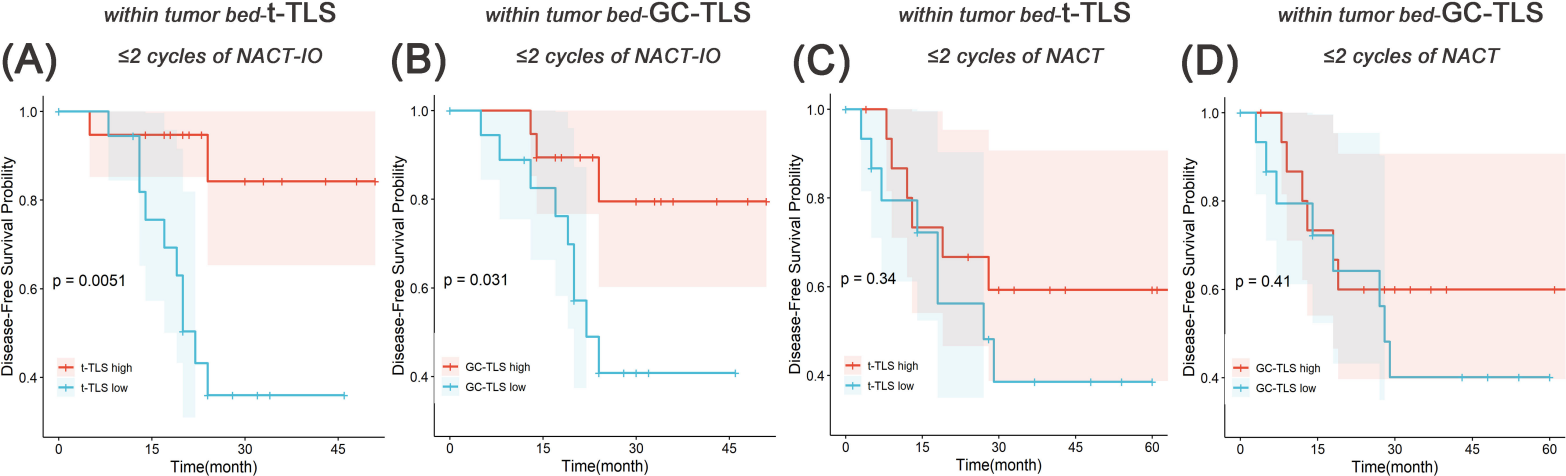
Survival analysis of t-TLS/GC-TLS in tumor bed in receiving ≤2 cycles of NACT-IO/NACT. The Kaplan-Meier curves of t-TLS **(A)** /GC-TLS **(B)** in the tumor bed high-/low- subgroup receiving ≤2 cycles of NACT-IO (n=38). The Kaplan-Meier curves of t-TLS **(C)** /GC-TLS **(D)** in the tumor bed high-/low- subgroup receiving ≤2 cycles of NACT (n=32). *p* < 0.05 was considered statistically significant.

The median follow-up duration was 24 months (range one day to 67 months). Based on the previously discovered treatment cycle-dependent immune microenvironment remodeling, we further verified whether TLS can be used as a biomarker to predict the survival benefit of combined therapy in a short period (≤ 2 treatment cycles) through survival analysis. According to the density distribution of t-/GC-TLS in the tumor bed of patients receiving ≤ 2 NACT-IO treatment cycles, the median value (median of t-TLS=0.37/mm^2^, range: 0.04/mm^2^-0.73/mm^2^; median of GC-TLS=0.14/mm^2^, range: 0/mm^2^-0.56/mm^2^) patients were divided into high/low group. Survival analysis results revealed that a significantly improved DFS in patients with high density t-/GC-TLS within the tumor bed (*p* = 0.005, *p* = 0.031) in patients who received ≤ 2 cycles of NACT-IO (n=38) (**Figures 7A, B**). However, the t-/GC-TLSs are not a prognostic factor for short-term (≤ 2 treatment cycles) NACT cases (n=32) (**Figures 7C, D**). In order to establish a clinically applicable TLS evaluation standard, this study stratified the t-/GC-TLS density in the tumor bed according to the threshold of hot spot area (t-TLS ≤/> 3/20×HPF, GC-TLS ≤/> 2/20×HPF). Multivariate analysis confirmed that the threshold of GC-TLS (≤ 2/20×HPF) could independently predict the survival risk of patients with ≤ 2 cycles of NACT-IO (HR = 3.99, 95%CI=1.10~14.5, *p* = 0.036), suggesting its potential as an auxiliary marker for treatment decision-making (**Table 2**).

**Table 2:** Univariate and multivariate analyses of DFS in patients with ≤ 2 cycles of NACT-IO (n=38).

Variable	Univariate		Multivariate	
	HR (95% CI)	*p*-value	HR (95% CI)	*p*-value
Age	1.57	0.44		
≥57 vs <57	(0.50 - 4.98)			
Gender	0.85	0.88		
male vs female	(0.11 - 6.84)			
Smoke history	1.53	0.53		
yes vs no	(0.41 - 5.65)			
N stage	1.33-3.51			
pN1 vs pN0	(0.28 - 6.46)	0.72		
pN2 vs pN0	(0.88 - 13.92)	0.07		
Number of t-TLS/20×HPF	4.35	0.013	2.88	0.11
≤3 vs >3	(1.37 - 13.8)		(0.78 - 10.56)	
Number of GC-TLS/20×HPF	6.70	<0.01	3.99	0.036
≤2 vs >2	(2.06 - 21.76)		(1.10 - 14.5)	
Pathologic response	3.05	0.05	3.44	0.044
PPR vs CPR/MPR	(0.98 - 9.49)		(1.03 - 11.47)	

DFS, disease-free survival; HR, hazard ratio; CI, confidence interval; TLS, tertiary lymphoid structures; CPR, complete pathological response; MPR, major pathological response; PPR, partial pathological response.

### Reproducibility of assessing the t-TLS/GC-TLS within tumor bed hotspots quantification

Independent assessment by two pathologists demonstrated both near-perfect agreement in t-TLS hotspot identification (Cohen’s κ = 0.85, 95% CI: 0.70 - 0.95). and GC-TLS hotspot identification (Cohen’s κ = 0.81, 95% CI: 0.62 - 0.94), confirming the operational robustness of this metric in routine pathology practice.

## Discussion

TIME is rich in components, but a large number of studies have focused on the T lymphocyte subsets (23). The CD8^+^T cells are highly destructive anti-tumor immune effector cells. Chemotherapy-induced immunogenic cell death (ICD) promotes the maturation and activation of dendritic cells (DCs), enabling them to present tumor antigens to CD8^+^T cells, thereby activating specific cytotoxic T lymphocyte responses (24). The combination of chemotherapy and immune checkpoint inhibitors can enhance this anti-tumor effect (25). We observed by mIF that CD8^+^T cells increased in most cases after receiving NACT/NACT-IO, even if not significantly. In recent years, various clinical studies of anti-tumor immune cells are rapidly expanding beyond the T cell-centered perspective, especially B cells (26). B cells could play an active role in anti-tumor immunity by acting as antigen-presenting cells, participating in the formation of TLS, and producing antibodies (27). We observed that the common role of NACT and NACT-IO is to significantly promote the infiltration of CD19^+^B cells and participate in the remodeling of TIME in SCC. The results of the study on NSCLC patients after neoadjuvant chemotherapy show that the prognosis of patients with SCC is not only affected by T cells, but also related to B cells, which is different from adenocarcinoma (28). B lymphocytes are the main components of TLS, and T cells are also one of the indispensable components (21, 29, 30). As an ‘ *in situ* immune factory ‘, TLS supports the differentiation of B cells into plasma cells (antibody secretion) and memory B cells through germinal center, and promotes the synergistic killing of Tfh-CD8^+^T cells (30). Therefore, B cells may be a key factor involved in the neoadjuvant therapy of SCC (chemotherapy alone or combined immunotherapy), which may provide a cellular basis for the formation of TLS.

The presence of the TLS structure often indicates a good prognosis outcome of the tumor (9, 21, 29), and it can also significantly enhance the anti-tumor effect of neoadjuvant immunochemotherapy by integrating local adaptive immune responses and systemic inflammatory signals (31). In fact, there is a ‘ two-way interaction ‘ between TLS and clinical treatment measures. In clinical practice, vaccines (32) and cisplatin (33) have been found to induce TLS in high-grade cervical intraepithelial neoplasia and hepatoblastoma. Therefore, further study on the induction of TLS by which drugs and treatment methods will help to develop new cancer treatment strategies to improve prognosis and survival rate. In this study, the results of cross-sectional comparative analysis among the three groups revealed the mechanism of synergistic regulation of TLS generation between chemotherapy and immunotherapy. In fact, in addition to maturity, the localization pattern has also been proved to be one of the key features of TLS functional differentiation. Patients with TLS in hepatocellular carcinoma (HCC) have a significantly lower risk of early recurrence within 2 years after surgery. However, there was no significant correlation between the density or maturity of TLS located at the edge of HCC or surrounding non-cancerous tissues and the risk of early recurrence (14). Interestingly, patients with cholangiocarcinoma (16) and esophageal squamous cell carcinoma (19) with high density of TLS around the tumor benefited significantly from chemotherapy combined with immunotherapy. In addition, another study of breast cancer suggested that patients with high TLS density around the tumor had a lower response rate to neoadjuvant chemotherapy (15). In this study, subgroup analysis based on spatial topological features confirmed that NACT/NACT-IO can induce TLS to show specific enrichment in the high exposure area of tumor antigen (the area where the original tumor tissue exists, that is, in the tumor bed), suggesting that the synergy of chemotherapy and immunotherapy drives the ecological position of TLS directional recruitment in the tumor bed. Few studies have explored to investigate the exact relationship between spatial distribution of TLS and clinical prognosis in SCC. To the best of our knowledge, this study represents the first attempt to assess the spatial aspects of TLS in SCC.

Silia et al. found that chemotherapy combined with corticosteroid in the treatment of lung squamous cell carcinoma could inhibit the formation of GC, a key functional unit in TLS, and weaken its anti-tumor potential (11). Zhang ‘s team confirmed in bladder cancer that platinum-based chemotherapy synergistically promotes TLS regeneration and maturation by inducing tumor immunogenic death to release antigens, activating dendritic cells, and combining programmed cell death 1/programmed cell death ligand 1(PD - 1/PD-L1) inhibitors (22). The analysis of the three groups and the analysis of the spatial sub-component layer in this study also supported that platinum-based chemotherapy alone and combined immunotherapy could significantly improve the mature phenotype differentiation of TLS, which may suggest that the immune regulation role of chemotherapy has the characteristics of ‘ treatment combination dependence ‘. Prolonging the cycle of chemotherapy combined with immunotherapy to 3 – 4 cycles can significantly achieve higher MPR rate (34). In fact, the extension of the treatment cycle may lead to delayed surgery, but short-term treatment may not be enough to maximize the effect of immunotherapy. The maturation of TLS requires continuous antigen exposure and immune microenvironment remodeling, which also suggests that the number of treatment cycles may directly affect its functional maturity and spatial distribution. Our results show that the promotion effect of NACT on GC is significantly weakened after the extension of the cycle. Interestingly, the generation of GC in TLS was sustained after combined immunotherapy, and the prolongation of the treatment cycle did not significantly weaken this effect. On the one hand, we confirmed that long-term NACT treatment can lead to the depletion of TLS and weakening of TLS maturity, and on the other hand it also revealed chemotherapy combined immunotherapy may antagonize negative regulation by maintaining the function of TLS. It has been reported that after receiving one NAC cycle, the abundance of CD8^+^T cells in the tumor of breast cancer patients can be significantly increased (35). Our data show that after long-term NACT treatment, CD8^+^T cells changed from ‘ functional enrichment ‘ to ‘ ineffective dispersion ‘. However, the functional integrity and maturity of TLS are still maintained after prolonging the cycle of NACT-IO, and CD8^+^T cells located in GC-TLS are anchored by regulating the homeostasis of immune microenvironment. This also suggests that the ‘ double-edged sword ‘ effect of early pro-inflammatory and late immunosuppression of simple chemotherapy may directly destroy the functional coupling of TLS-CD8^+^T cells. This will make it difficult to determine a time window to reach the balance point of tumor immune tolerance.

The TLS, particularly those harboring GC formations, serves as a robust independent predictor of therapeutic benefit in solid tumors. In patients with NSCLC who received neoadjuvant therapy, TLS infiltration was significantly increased in MPR or pCR compared with non-MPR or non-pCR (36). And, the GC-TLSs are positively correlated with MPR rate in NSCLC (37). When exploring TLS and treatment benefits, we found that the t-TLS of CPR/MPR patients in the NACT-IO group was significantly better than that of PPR patients, not GC-TLS. The t-TLS and GC-TLS of PPR patients in the NACT group were not only slightly higher than those in the UT group (baseline), but also higher than those in the CPR/MPR patients. The TLS acts as a local immune niche to promote adaptive immunity, and its non-encapsulated structure can be directly exposed to various stimuli in TIME (38). The presence of tumor specific antigen is one of the factors for the formation of GC in TLS (9). GC is an important site for the activation, proliferation and differentiation of B cells into antibody-secreting cells in TLS (39). Therefore, we speculate that the formation and maturation of TLS induced by NACT may be largely dependent on tumor tissue load. When the tumor volume is significantly reduced, the antigen release is reduced, and t-TLS and GC-TLS are degraded due to lack of continuous stimulation. Although the combined immunotherapy may maintain the compensatory proliferation of TLS, it is not enough to continue to activate GC. These findings provide a more detailed idea for mining TLS-related indicators as prognostic indicators for SCC patients receiving neoadjuvant therapy.

Finally, this study innovatively developed a spatial hotspot counting method based on digital pathological images. By quantifying the aggregation distribution characteristics of TLS in the tumor bed (rather than simply counting), it revealed the strong correlation between TLS spatial topological pattern and DFS for the first time. Survival analysis of patients receiving ≤ 2 cycles of NACT-IO showed that the DFS rate of patients in the high t-/GC-TLS group was significantly better than that in the low t-/GC-TLS group. Further, the hot spot assessment of t-TLS (HR = 4.35, 95%CI: 1.37 - 13.8, *p* = 0.013) and GC-TLS (HR = 6.70, 95%CI: 2.06 - 21.76, *p* < 0.01) in univariate analysis was strongly correlated with DFS benefit. It is worth noting that in the multivariate Cox regression model, GC-TLS hotspot assessment was independent of the traditional prognostic factor of pathological remission (HR = 3.99, 95%CI=1.10-14.5, *p* = 0.036) and became the strongest independent factor for predicting DFS. This finding breaks through the limitations of traditional TLS semi-quantitative evaluation, revealing that spatial clustering GC-TLS (rather than isolated scattered distribution) is an important feature of clinical benefits, and provides a quantifiable new biomarker system for optimizing the stratification of patients receiving NACT-IO.

## Conclusions and prospects

This study analyzed the dynamic regulatory network of neoadjuvant therapy (chemotherapy ± immune checkpoint inhibitors) on TLS, a key hub of TIME in SCC, and clarified that the treatment mode (chemotherapy alone vs combined immunization) and cycle number differences reshape TLS density, spatial topology and functional maturity (GC formation) through a time-dependent mechanism. For the first time, we quantified the spatial aggregation characteristics of TLS (hot spot assessment method) based on digital pathological images, and further explored multi-dimensional topological biomarkers for predicting disease progression, providing a clinically practical and repeatable evaluation tool for optimizing SCC neoadjuvant treatment decisions. However, the study still has the following limitations: Firstly, the sample size is small and is a retrospective design, which may affect the statistical effectiveness. Secondly, the functional verification of GC-TLS (such as spatial transcriptome or TCR clonality analysis) has not yet been deepened, and its regulatory mechanism needs further analysis. Thirdly, the causal relationship between treatment cycle and TLS dynamics needs to be verified by prospective studies.

## Data Availability

The raw data supporting the conclusions of this article will be made available by the authors, without undue reservation.
