# Correction: The germinal center-tertiary lymphoid structure after neoadjuvant chemo-immunotherapy for locally advanced lung squamous cell carcinoma can predict the disease progression

**DOI:** 10.3389/fimmu.2025.1721980

**Published:** 2025-10-20

**Authors:** Shuang Li, Ping Zhou, Yan Huang, Min Chen, Chan Yang, Lili Jiang

**Affiliations:** ^1^ Department of Pathology, West China Hospital, Sichuan University, Chengdu, China; ^2^ Department of Pathology, The First People’s Hospital of Yunnan Province, Yunnan, Kunming, China; ^3^ Department of Medical Oncology, Cancer Center, West China Hospital, Sichuan University, Chengdu, Sichuan, China; ^4^ College of Physical Education and Health Science, Yi bin University, Yi bin, Sichuan, China

**Keywords:** neoadjuvant chemoimmunotherapy, lung squamous cell carcinoma, prognostic biomarkers, tertiary lymphoid structures (TLSs), neoadjuvant chemotherapy

In the published article, there was an error in the author list, and author “Shuang Li” was erroneously sorted. The corrected author list appears below.

“Shuang Li ^1,2^, Ping Zhou^1^, Yan Huang^3,4^, Min Chen^1^, Chan Yang^1^ and Lili Jiang^1^*”

The authors apologize for this error and state that this does not change the scientific conclusions of the article in any way. The original article has been updated.

