# Supplementary material for: The germinal center-tertiary lymphoid structure after neoadjuvant chemo-immunotherapy for locally advanced lung squamous cell carcinoma can predict the disease progression
Source: Front Immunol. 2025 Sep 12;16:1579840. doi: 10.3389/fimmu.2025.1579840 (PMC12463958; doi:10.3389/fimmu.2025.1579840)
Supplement: Supplementary file 1 [file Table1.docx]

| Antibody | Cloning number | Manufacturer | CatalogNumber | Dilution rate | Usage |
| --- | --- | --- | --- | --- | --- |
| CD4 | UMAB64 | ZSGB-Bio | ZM-0418 | 1:100 | mIF |
| CD8 | SP16 | ZSGB-Bio | ZA-0508 | 1:100 | mIF |
| CD19 | UMAB103 | ZSGB-Bio | ZM-0038 | 1:100 | mIF |
| PD1 | UMAB199 | ZSGB-Bio | ZM-0381 | 1:100 | mIF |
| CD8 | SP16 | ZSGB-Bio | ZA-0508 | 1:100 | IHC |
| CD19 | BT51E | Univ | BT51E | 1:100 | IHC |
| CD21 | EP64 | MXB | RMA-0811 | 1:100 | IHC |
| CD23 | EP75 | ZSGB-Bio | ZM-0516 | 1:100 | IHC |
| D2-40 | D2-40 | ZSGB-Bio | ZM-0465 | 1:100 | IHC |

**Antibody Lot-to-Lot Validation Protocol**

(a). Historical data comparison. According to the titer conditions verified for the first time, the comparative staining was carried out to observe whether the new batch of antibodies was consistent with the previous batch in terms of staining intensity and specificity.

(b). Control tissue retest : Staining and recording were performed using preserved sections, and the staining results of the same control tissue by the new and old batches of antibodies were compared, including the positive rate of staining, staining intensity, staining location, etc.

(c). Gradient dilution adjustment : If the results of the old and new batches do not match, the antibody concentration or scheme needs to be re-optimized. By gradiently diluting the antibody, the concentration that can make the staining result reach the best state is found to ensure that the new batch of antibody can stably produce the expected staining effect at different concentrations.
