# Supplementary material for: The germinal center-tertiary lymphoid structure after neoadjuvant chemo-immunotherapy for locally advanced lung squamous cell carcinoma can predict the disease progression
Source: Front Immunol. 2025 Sep 12;16:1579840. doi: 10.3389/fimmu.2025.1579840 (PMC12463958; doi:10.3389/fimmu.2025.1579840)
Supplement: Supplementary file 2 [file Table2.docx]

Table 1. The t-TLS burden within tumor bed hotspots (binary: ≤3/>3/20×HPF)

| ID-patient | PathologistA | PathologistB |
| --- | --- | --- |
| Z1804534 | 1 | 1 |
| Z1825771 | 0 | 0 |
| Z1835946 | 1 | 1 |
| Z1847555 | 1 | 1 |
| Z1852063 | 1 | 1 |
| Z1860246 | 1 | 1 |
| Z1908655 | 0 | 0 |
| Z1910517 | 1 | 1 |
| Z1915839 | 1 | 1 |
| Z1926482 | 1 | 1 |
| Z1951076 | 1 | 1 |
| Z1967941 | 1 | 1 |
| Z2006828 | 0 | 0 |
| Z2008463 | 1 | 1 |
| Z2018384 | 1 | 1 |
| Z2022120 | 1 | 1 |
| Z2036056 | 0 | 0 |
| Z2062114 | 1 | 1 |
| Z2062118 | 1 | 1 |
| Z2104613 | 1 | 1 |
| Z2152872 | 0 | 0 |
| Z2153631 | 1 | 1 |
| Z2153632 | 1 | 0 |
| Z2162300 | 0 | 1 |
| Z2209157 | 0 | 0 |
| Z2222250 | 0 | 0 |
| Z2222251 | 1 | 1 |
| Z2223433 | 0 | 0 |
| Z2225053 | 0 | 1 |
| Z2225345 | 0 | 0 |
| Z2227718 | 0 | 0 |
| Z2240659 | 1 | 1 |
| Z2260431 | 0 | 0 |
| Z2261045 | 1 | 0 |
| Z2261557 | 1 | 1 |
| Z2271289 | 1 | 1 |
| Z2274596 | 1 | 1 |
| Z2275292 | 1 | 1 |
| Z1817866 | 0 | 0 |
| Z1837170 | 0 | 0 |
| Z1838046 | 0 | 0 |
| Z1849513 | 0 | 0 |
| Z1910260 | 0 | 0 |
| Z1910754 | 0 | 0 |
| Z1911047 | 0 | 0 |
| Z1912525 | 0 | 0 |
| Z1913188 | 0 | 0 |
| Z1914640 | 1 | 0 |
| Z1917766 | 0 | 0 |
| Z1954443 | 0 | 0 |
| Z1955374 | 0 | 0 |
| Z1955662 | 0 | 0 |
| Z1956563 | 0 | 0 |
| Z1966789 | 0 | 0 |
| Z1969722 | 0 | 0 |
| Z2008079 | 0 | 0 |
| Z2008259 | 0 | 0 |
| Z2009594 | 0 | 0 |
| Z2016783 | 1 | 0 |
| Z2029623 | 0 | 0 |
| Z2033997 | 0 | 0 |
| Z2034562 | 0 | 0 |
| Z2036372 | 0 | 0 |
| Z2047834 | 0 | 0 |
| Z2049322 | 0 | 0 |
| Z2101868 | 0 | 0 |
| Z2103770 | 0 | 0 |
| Z2106467 | 0 | 0 |
| Z2109512 | 0 | 0 |
| Z2114999 | 0 | 0 |
| Z2117926 | 0 | 0 |
| Z2124032 | 0 | 0 |
| Z2133651 | 1 | 1 |
| Z2134009 | 0 | 0 |
| Z2152313 | 0 | 0 |
| Z2153913 | 0 | 0 |
| Z2160068 | 0 | 0 |
| Z2162518 | 0 | 0 |
| Z2164352 | 0 | 0 |
| Z2164616 | 0 | 0 |
| Z2165286 | 0 | 0 |
| Z2173595 | 0 | 0 |
| Z2212506 | 0 | 0 |
| Z2219815 | 0 | 0 |
| Z2232779 | 0 | 0 |
| Z2233187 | 0 | 0 |
| Z2233899 | 0 | 0 |
| Z2233907 | 0 | 0 |
| Z2237182 | 0 | 0 |
| Z2240664 | 0 | 0 |
| Z2242308 | 0 | 0 |
| Z2247464 | 0 | 0 |
| Z2248213 | 0 | 0 |
| Z2254124 | 0 | 0 |
| Z2255384 | 0 | 0 |
| Z2260156 | 0 | 0 |
| Z2262119 | 0 | 0 |
| Z2268592 | 0 | 0 |
| Z2284308 | 0 | 0 |
| Z2286417 | 0 | 0 |
| “0”denotes “>3/20×HPF”, “1”denotes “≤3/20×HPF” | | |

Table 2. The GC-TLS burden within tumor bed hotspots (binary: ≤2/>2/20×HPF)

| ID-patient | PathologistA | PathologistB |
| --- | --- | --- |
| Z1804534 | 1 | 1 |
| Z1825771 | 1 | 1 |
| Z1835946 | 0 | 0 |
| Z1847555 | 1 | 1 |
| Z1852063 | 0 | 0 |
| Z1860246 | 1 | 1 |
| Z1908655 | 0 | 0 |
| Z1910517 | 1 | 1 |
| Z1915839 | 1 | 1 |
| Z1926482 | 1 | 1 |
| Z1951076 | 1 | 1 |
| Z1967941 | 0 | 0 |
| Z2006828 | 0 | 0 |
| Z2008463 | 0 | 0 |
| Z2018384 | 1 | 1 |
| Z2022120 | 0 | 0 |
| Z2036056 | 0 | 0 |
| Z2062114 | 1 | 1 |
| Z2227718 | 1 | 0 |
| Z2240659 | 0 | 0 |
| Z2260431 | 1 | 1 |
| Z2261045 | 1 | 1 |
| Z2261557 | 0 | 0 |
| Z2271289 | 0 | 0 |
| Z2274596 | 1 | 1 |
| Z2275292 | 1 | 0 |
| Z1817866 | 1 | 1 |
| Z1837170 | 0 | 0 |
| Z1838046 | 0 | 0 |
| Z1849513 | 0 | 0 |
| Z1910260 | 0 | 0 |
| Z1910754 | 0 | 0 |
| Z1911047 | 0 | 0 |
| Z1912525 | 0 | 0 |
| Z1913188 | 0 | 0 |
| Z1914640 | 0 | 0 |
| Z1917766 | 0 | 0 |
| Z1935037 | 0 | 0 |
| Z1951078 | 0 | 0 |
| Z1954443 | 0 | 0 |
| Z1955374 | 0 | 0 |
| Z1955662 | 0 | 0 |
| Z1956563 | 0 | 0 |
| Z1966789 | 0 | 0 |
| Z1969722 | 0 | 0 |
| Z2008079 | 1 | 0 |
| Z2008259 | 1 | 1 |
| Z2009594 | 0 | 0 |
| Z2016783 | 0 | 0 |
| Z2029623 | 0 | 0 |
| Z2033997 | 0 | 0 |
| Z2034562 | 0 | 0 |
| Z2036372 | 0 | 0 |
| Z2047834 | 0 | 0 |
| Z2049322 | 0 | 0 |
| Z2101868 | 0 | 0 |
| Z2103770 | 0 | 0 |
| Z2106467 | 1 | 0 |
| Z2109512 | 0 | 0 |
| Z2114999 | 0 | 0 |
| Z2117926 | 0 | 0 |
| Z2124032 | 1 | 1 |
| Z2133651 | 1 | 1 |
| Z2134009 | 0 | 0 |
| Z2137414 | 0 | 0 |
| Z2137415 | 0 | 0 |
| Z2138237 | 0 | 0 |
| Z2142446 | 0 | 0 |
| Z2143585 | 0 | 0 |
| Z2143902 | 0 | 0 |
| Z2148141 | 0 | 0 |
| Z2148962 | 0 | 0 |
| Z2149956 | 0 | 0 |
| Z2152023 | 0 | 0 |
| Z2152313 | 0 | 0 |
| Z2153913 | 0 | 0 |
| Z2160068 | 0 | 0 |
| Z2162518 | 0 | 0 |
| Z2164352 | 0 | 0 |
| Z2164616 | 0 | 0 |
| Z2165286 | 0 | 1 |
| Z2173595 | 0 | 0 |
| Z2212506 | 0 | 0 |
| Z2219815 | 0 | 0 |
| Z2232779 | 0 | 0 |
| Z2233187 | 0 | 0 |
| Z2233899 | 0 | 0 |
| Z2233907 | 0 | 0 |
| Z2237182 | 0 | 0 |
| Z2240664 | 0 | 0 |
| Z2242308 | 0 | 1 |
| Z2247464 | 0 | 0 |
| Z2248213 | 0 | 0 |
| Z2254124 | 0 | 0 |
| Z2255384 | 0 | 0 |
| Z2260156 | 0 | 0 |
| Z2262119 | 0 | 0 |
| Z2268592 | 0 | 0 |
| Z2284308 | 0 | 0 |
| Z2286417 | 0 | 0 |
| “0”denotes “>2/20×HPF”, “1”denotes “≤2/20×HPF” | | |

Table 3.

|  | Pathologists B/t-TLS  (≤ 3/20×HPF) | Pathologists B/t-TLS  (>3/20×HPF) | totally |
| --- | --- | --- | --- |
|  |  |  |  |
| Pathologists A/t-TLS  (≤ 3/20×HPF) | 24 | 4 | 28 |
| Pathologists A/t-TLS  (>3/20×HPF) | 2 | 70 | 72 |
| totally | 26 | 74 | 100 |

Table 4.

|  | Pathologists B/GC-TLS  (≤ 2/20×HPF) | Pathologists B/GC-TLS  (>2/20×HPF) | totally |
| --- | --- | --- | --- |
|  |  |  |  |
| Pathologists A/GC-TLS  (≤ 2/20×HPF) | 17 | 4 | 21 |
| Pathologists A/GC-TLS  (>2/20×HPF) | 2 | 77 | 79 |
| totally | 19 | 81 | 100 |

Cohen's κ was derived by:

Quantifying observed agreement（*Po*）

Adjusting for expected chance agreement（*Pe*）

Using the formula “ κ=(*Po-Pe)/*1-*Pe ”*

This 'substantial-to-almost perfect' agreement (per Landis & Koch) reflects the method's operational robustness in clinical pathology practice.

The inter-observer agreement was quantified using Cohen's κ, with 95% confidence intervals estimated via bias-corrected and accelerated (BCa) bootstrap resampling (1000 iterations).
